# Evaluation of 3 distinct file systems in root canal retreatment: A retrospective cohort study

**DOI:** 10.1097/MD.0000000000044922

**Published:** 2025-10-10

**Authors:** Qi Fang, Qianpeng Li, Yajun Gao, Yao Wang

**Affiliations:** a Department of Endodontics, Wuxi Stomatological Hospital, Wuxi, China; b Department of Oral Health Sciences, BIOMAT and UZ Leuven, Dentistry, KU Leuven, Leuven, Belgium; c Department of Oral Implantology, WuXi Stomatological Hospital, Wuxi, China.

**Keywords:** endodontics, root canal retreatment

## Abstract

This study aimed to evaluate the efficacy of 2 retreatment file systems, ROTATE Retreatment (RR) and Re-Treaty, in comparison with the general system (M3) for root canal retreatment. A retrospective controlled study was conducted involving 81 patients who underwent root canal retreatment using the M3, RR, or Re-Treaty systems. The operation times were recorded and analyzed. Pain intensity was assessed using the visual analog scale at 4 time points: preoperation (T0), 1 day postoperatively (T1), 7 days postoperatively (T2), and 3 months postoperatively (T3). Radiographic data were collected at T0 and T3, and lesion diameter and density were evaluated by 2 independent researchers. The final clinical outcomes were assessed at 1-year follow-up (T4). Compared to the Re-Treaty and M3 systems, the RR system significantly reduced operative time (*P* < .05). Success rates were high across all groups (M3, 88.9%; RR, 100%; Re-Treaty, 92.6%), with no statistically significant differences. RR has shorter operative time (13.15 ± 2.5 minutes/ canal) compared to M3 (15.93 ± 4.03 minutes/ canal) and Re-Treaty (15.04 ± 3.5 minutes/ canal) (*P* < .05). All systems effectively reduced patient pain in both the short- and long-term and promoted radiographic healing. The differences between the file systems were not statistically significant (*P* = .086). All 3 endodontic file systems achieved optimal retreatment outcomes and exhibited similar prognoses. The RR system had the shortest operative time.

## 1. Introduction

Nonsurgical root canal retreatment (RCT-R) is the most common approach used to address the failure of initial root canal treatment (RCT).^[1]^ The primary cause of RCT failure is persistent microbial infection in the root canal system and/or periapical region, which may arise due to inadequate cleaning and shaping, incomplete obturation, or coronal leakage that allows bacterial penetration into the root canal system.^[2]^ These factors can lead to persistent periapical lesions and symptoms, necessitating retreatment to achieve microbial control and periapical healing.

General-purpose endodontic file systems commonly used in clinical practice are typically designed with triangular, elongated rectangular, rectangular, and square cross-sections, and feature a non-cutting safety tip. This design is effective for cutting dentin while maintaining its strength and preventing lateral perforations. Thus, these universal file systems are suitable for a wide range of clinical applications including retreatment.

However, the first step of RCT-R is to remove the existing filling materials. These universal file systems do not perform well when cutting root canal filling materials, typically gutta-percha.^[3]^ This can lead to residual gutta-percha on the root canal walls or a prolonged treatment time because of low cutting efficiency. To overcome this issue, some manufacturers have introduced specialized root canal file systems that are specifically designed for retreatment. These new systems often feature unique cross-sectional structures and cutting tips that enhance the efficiency of re-preparation.

In our study, the experimental group utilized 2 retreatment-specific endodontic file systems: the ROTATE™ Retreatment (RR) system (VDW, Germany) and the Re-Treaty system (Perfect, China). Compared to the control group, the RR system features an improved tip and an S-shaped cross-section, enhancing its ability to engage and remove gutta-percha more effectively.^[4,5]^ Re-Treaty is another root canal file system that incorporates 5 nickel-titanium (NiTi) files. It has an S-shaped cross-section, is made of heat-treated gold alloy, and features a shorter apical thread.

In contrast, we used the M3 universal NiTi rotary file system (United Dental Group, China) as the control group because it features a traditional convex triangular or square cross section. This cross-sectional design provides resistance to torsional stress and prevents instrument fracture, and is widely used and suitable for various clinical scenarios.

While numerous in vitro studies have assessed various aspects of root canal file systems, such as cutting efficiency on gutta-percha, debris extrusion, apical transportation, and centering ability,^[6–10]^ there is a lack of clinical evidence to determine whether these findings translate into meaningful outcomes in real-world practice.

This clinical study included 81 patients and assessed both short-term and long-term patient-reported pain levels using the Visual Analog Scale (VAS), which is typically presented as a horizontal line anchored with 2 verbal descriptors at the extremes, where respondents indicate their pain status by placing a mark along the line at the most appropriate point.^[11]^ We also analyzed posttreatment radiographic changes in the lesions to evaluate the healing rate of periapical tissue.

This study aimed to evaluate and compare the efficacy of 3 different file systems in root canal retreatment, providing theoretical guidance for clinicians and supporting informed decision making in the selection of retreatment file systems.

## 2. Methods and materials

**Figure 1.**
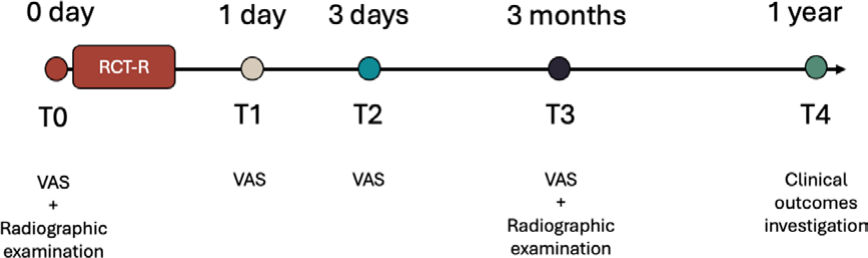
Timeline of the study protocol.

This retrospective study was approved by the Ethics Committee of Wuxi Stomatological Hospital (No. 2025010204). A total of 81 patients who attended the Department of Endodontics between March 2022 and February 2023 were included, with 27 participants assigned to each group. All participants provided written informed consent after receiving information about the treatment procedures. The flowchart shows the timeline of study (Fig. 1).

### 2.1. Cases selection

Specific inclusion and exclusion criteria were established for patient selection to ensure the reliability and relevance of this study. The inclusion and exclusion criteria were as follows:

### 2.2. Inclusion criteria

1. Patients aged between 18 and 70 years with generally good health or well-controlled systemic conditions (e.g., hypertension and diabetes).
2. At least one previously treated root canal showed signs of failure such as persistent symptoms, radiographic evidence of inadequate filling, or periapical radiolucency.
3. Radiographic (X-ray or CBCT) confirmation of a periapical lesion with a radiolucency > 2 mm.
4. Clinical signs of periapical inflammation, including tenderness upon palpation, percussion pain, or sinus tract formation, but no acute periapical abscess requiring emergency intervention.
5. Teeth with fully formed roots and no signs of active periodontal disease or severe mobility.
6. Patients able and willing to provide informed consent for the study.
7. Teeth with no complex anatomical abnormalities or extensive resorptive lesions visible on radiographic evaluation.

### 2.3. Exclusion criteria

1. Patients with systemic diseases or conditions contraindicating endodontic treatment (e.g., uncontrolled diabetes, immunosuppression).
2. Pregnant or breastfeeding women.
3. Teeth with severe coronal destruction, insufficient to support a restorative procedure posttreatment.
4. Previous retreatment of the affected tooth or presence of metal obstructions (e.g., posts) that prevent instrumentation with the selected file systems.
5. Patients unable to comply with follow-up visits or instructions for postoperative care.

### 2.4. Sample calculation

The sample size was calculated using G*Power 3.1.9.2, based on a previous study^[12]^ and the VAS score served as the primary outcome parameter. In that study, the mean VAS scores for the 2 groups were 2.57 ± 1.22 and 1.62 ± 0.92, respectively, indicating a clinically relevant difference in posttreatment pain levels. Using these values as a reference and setting *α* = 0.05, and 1-*β* = 80%, a minimum of 24 patients per group was determined to be sufficient to detect a statistically significant inter-group difference (*P* = .05) using a 2-tailed t-test with a statistical power of 81.3%. This calculation ensured adequate sensitivity to evaluate the effectiveness of the intervention based on patient-reported pain levels.

### 2.5. Root canal re-preparation

All procedures were performed by the same experienced operator to minimize inter-operator variability. A standardized protocol was strictly followed for each instrument system, including working length determination, rotational speed, torque setting, irrigation volume, and sequence of file use. The detailed procedure is described as follows:

Radiographs were taken to assess the root canal system, including canal position, width, length, and curvature. The original filling materials and carious tissues were removed, and the pulp chamber was inspected for any missing canals. The rubber dam was then isolated.

For M3, the upper portion of the root canal was prepared using an orifice opener (17/.08) at 300 rpm and 3.0 N·cm torque. A pathway was established with a pathfinding file (19/.02) at 350 rpm and 1.5 N·cm torque. Sequential preparation was then carried out using the files 15/.06 (350 rpm, 2.0 N·cm), 25/.04 (350 rpm, 1.5 N·cm), 25/.06 (350 rpm, 2.0 N·cm), and 35/.04 (350 rpm, 1.5 N·cm).

For RR, gutta-percha was removed using the RR 25/.05 file (400 rpm, 3.5 N·cm) with an upward pulling motion. Canal preparation was performed on the working length using the RR 25/.06 file (400 rpm, 2.3 N·cm).

For Re-treaty, Gutta-percha removal was initiated using the BullY 25/.07 instrument operated at 400 rpm and 1.5 N·cm torque, targeting the coronal 3 to 4 mm of the canal with a brushing motion to remove bulk filling material. This was followed by SkinnY 25/.04 at 400 rpm, 1.5 N·cm, used to clean the middle third of the canal and advance apically. To establish patency and reach the working length, a SkinnY1 20/.04 file was introduced using a gentle pecking motion. Once working length was confirmed, sequential canal shaping was performed using ShapY2 (400 rpm, 1.0 N·cm), followed by 2 cycles of ShapY3 (400 rpm, 1.5 N·cm) to complete final preparation.

Each file change was accompanied by canal irrigation with 3% sodium hypochlorite and 17% EDTA. Ultrasonic irrigants were used to clean the root canals.

### 2.6. Obturation

The canals were thoroughly irrigated with 3% sodium hypochlorite and 17% EDTA to remove debris and smear layers. Final irrigation was performed using saline to neutralize the canal environment. The canals were dried using sterile paper points until no moisture was visible.

The single-cone technique combined with warm vertical compaction was used for obturation. A gutta-percha cone matching the size and taper of the final instrumented canal was selected and its fit was verified to the working length. The cone was coated with an iRoot® sp root canal sealer and carefully placed in the canal. Warm vertical compaction was performed using a heated plugger to soften the gutta-percha and ensure that it adapted well to the canal walls, filling all irregularities and accessory canals. Additional segments of gutta-percha were added and vertically compacted until the canal was completely obturated.

After obturation, a radiograph was obtained to verify the quality of the root canal filling. The tooth was temporarily or permanently restored.

### 2.7. Data collection

At the patient’s initial visit, general information including tooth position, sex, and age was recorded. Root canal retreatment was performed following the standardized protocol outlined in the previous section, and operative time was documented. Follow-up assessments were conducted at specific time points: before treatment (T0), 24 hours posttreatment (T1), 72 hours (T2), 3 months (T3), and 1-year (T4) posttreatment.

Patients evaluated their Pain levels using a 0 to 10 VAS, where 0 indicated no pain and 10 represented the most severe pain imaginable. Evaluations were performed at predetermined time intervals (T0, T1, T2, and T3). To ensure reliability, patients were given standardized instructions on how to use the VAS before treatment, and any uncertainties were clarified.

Periapical radiographs were obtained at T0 and T3 for all patients using Heliodent Plus D3507 (Sirona Dental Systems, Germany). For all patients, the radiographic parameters were set at 60 kVp, 6 mA, and 0.05 seconds. Radiographic length calibration was performed using a reference metal ball of known diameter, which was placed adjacent to the region of interest during image acquisition. Linear measurements were subsequently adjusted based on this calibration using ImageJ software.

The relative radiographic density of the lesions was calculated by comparing the radiographic density of the apical lesion to that of the surrounding normal bone tissue using ImageJ software as follows:


$$\begin{array}{l} RRD=\frac{G_{L}}{G_{N}} \end{array}$$

*RRD* = Relative Radiographic Density

*G*_*L*_ = Mean Gray Value of the Les ion Area

*G*_*N*_ = Mean Gray Value of the Normal Tissue

To minimize bias, all measurements were independently performed by 2 blinded, calibrated examiners who were not involved in the treatment procedure. Intraexaminer reliability was confirmed through intraclass correlation coefficient analysis using a 2-way random-effects model. Consistency was considered excellent (intraclass correlation coefficient > 0.9). In cases of discrepancies between reported values, the mean value was used for the final analysis.

All patients were followed-up at 1-year posttreatment (T4) to assess the clinical outcomes of the treated teeth.

### 2.8. Statistical analysis

Data analysis was performed using the Statistical Package for the Social Sciences (IBM SPSS Statistics, version 28, USA). Unless otherwise specified, all continuous variables were presented as mean ± standard deviation. Continuous variables were assessed for normality using the Shapiro–Wilk test and for homogeneity of variance using Levene test. Data conforming to normal distribution and equal variances were analyzed using the independent samples t-test or one-way ANOVA, as appropriate. When the assumption of equal variances was not met, Welch t-test or Welch ANOVA was employed. For non-normally distributed data, the Mann–Whitney *U* test or Kruskal–Wallis test was used. A 2-sided *P*-value <.05 was considered statistically significant. Categorical variables were compared using the chi-square test.

## 3. Results

### 3.1. General information

The study included 81 patients, with 27 patients undergoing RCT-R using the M3 file system, 27 using the RR system, and 27 using the Re-Treaty file system. The demographic characteristics of the patients and types of teeth treated are shown in Table 1. Statistical analysis using the chi-square test revealed no significant differences in age, sex, or tooth position among the 3 groups (*P* > .05). During the entire study period, there were no cases of missing data or loss to follow-up among the patients.

**Table 1:** Demographic features of the patients

	M3	RR	Re-treaty	*P*-value (asymp. sig.)
N				
	27	27	27	–
Gender				
Male	10 (37.0%)	9 (33.3%)	16 (59.3%)	.115
Female	17 (63.0%)	18 (66.7%)	11 (40.7%)	
Age				
≤35 yr old	15 (56%)	13 (48%)	10 (37%)	.390
>35 yr old	12 (44%)	14 (52%)	17 (63 %)	
Tooth number				
Anterior teeth	11 (41%)	7 (26%)	12 (44%)	.602
Premolars	7 (26%)	9 (33%)	5 (19%)	
Molars	9 (33%)	11 (41%)	10 (37%)	

RR = ROTATE™ retreatment.

### 3.2. Operative duration and treatment outcomes

The operative times for the 3 file systems are listed in Table 2. The RR system demonstrated the shortest operative time (13.15 ± 2.5 minutes), which was significantly lower than that of the control group (*P* < .05). While the Re-Treaty system also had a shorter mean operative time than the control group, the difference was not statistically significant (*P* > .05).

**Table 2:** Operative time.

Mean ± SD (min/canal)	M3	RR	Re-treaty
Operative time	15.93 ± 4.03	13.15 ± 2.5	15.04 ± 3.5
*P*-value (vs M3)	/	.004*	.34

SD = standard deviation.

*P < .05.

The 1-year postoperative outcomes in each group are summarized in Table 3. In this study, successful retreatment was defined as the absence of symptoms, recurrence or enlargement of apical lesions, and radiographic evidence of periapical healing or stability.

**Table 3:** Treatment outcomes 1-yr postoperatively.

n (%)	M3	RR	Re-treaty	*P*-value (asymp. sig.)
Success	24 (88.9%)	27 (100%)	25 (92.6%)	.53
Tooth extraction	1 (3.7 %)	0	1 (3.7 %)	
Retreatment	2 (7.4%)	0	1 (3.7 %)	

In the RR group, all patients (100%) achieved successful outcomes, with no patients requiring tooth extraction or further retreatment. In the Re-Treaty group, the success rate was 92.6% (25 cases), with 1 patient requiring tooth extraction and 1 patient (3.7%) requiring retreatment. In the M3 group, the success rate was 88.9% (24 cases), with 1 patient undergoing tooth extraction and 2 patients requiring retreatment.

Chi-square tests revealed no statistically significant differences in treatment outcomes among the 3 groups at the 1-year follow-up (*P* = .53). These findings indicate that the M3, RR, and Re-Treaty file systems exhibited comparable success rates in root canal retreatment, with no significant differences observed.

### 3.3. VAS

**Figure 2.**
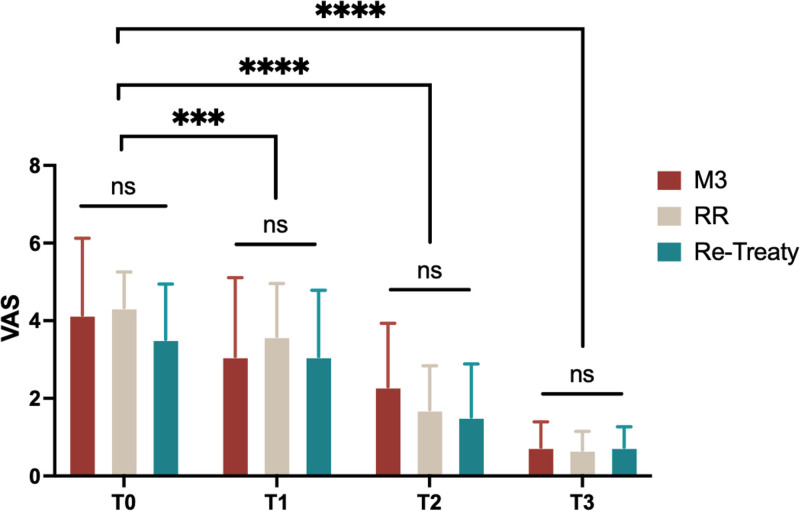
The VAS values were reported by patients at different time points. Values are expressed as means ± SD. ***P < .001, ****P < .0001. ns = not significant, SD = standard deviation, VAS = visual analog scale.

The VAS scores are presented in Table 4 and Figure 2. No significant differences were observed in the initial VAS score (T0) among the 3 groups (*P* > .05). All 3 groups demonstrated a time-dependent decrease in the VAS values, and the difference was statistically significant (*P* < .0001). However, during all postoperative follow-ups, no significant differences were observed between the groups at any time point (T1, T2, and T3).

**Table 4:** VAS reported by patients.

VAS (Mean ± SD)	M3	RR	Re-treaty	*P*-value
n	27	27	27	–
T0	4.15 ± 1.98	4.33 ± 0.92	3.52 ± 1.42	.12
Preoperative follow-up				
T1	3.07 ± 2.04	3.59 ± 1.37	3.07 ± 1.71	.45
T2	2.30 ± 1.64	1.70 ± 1.14	1.52 ± 1.37	.11
T3	0.74 ± 0.66	0.67 ± 0.48	0.74 ± 0.53	.85
*P*-value (one-way Anova)	<.0002*	<.0001*	<.0001*	–

VAS = visual analog scale.

*P < .05.

### 3.4. Radiographic analysis

**Figure 3.**
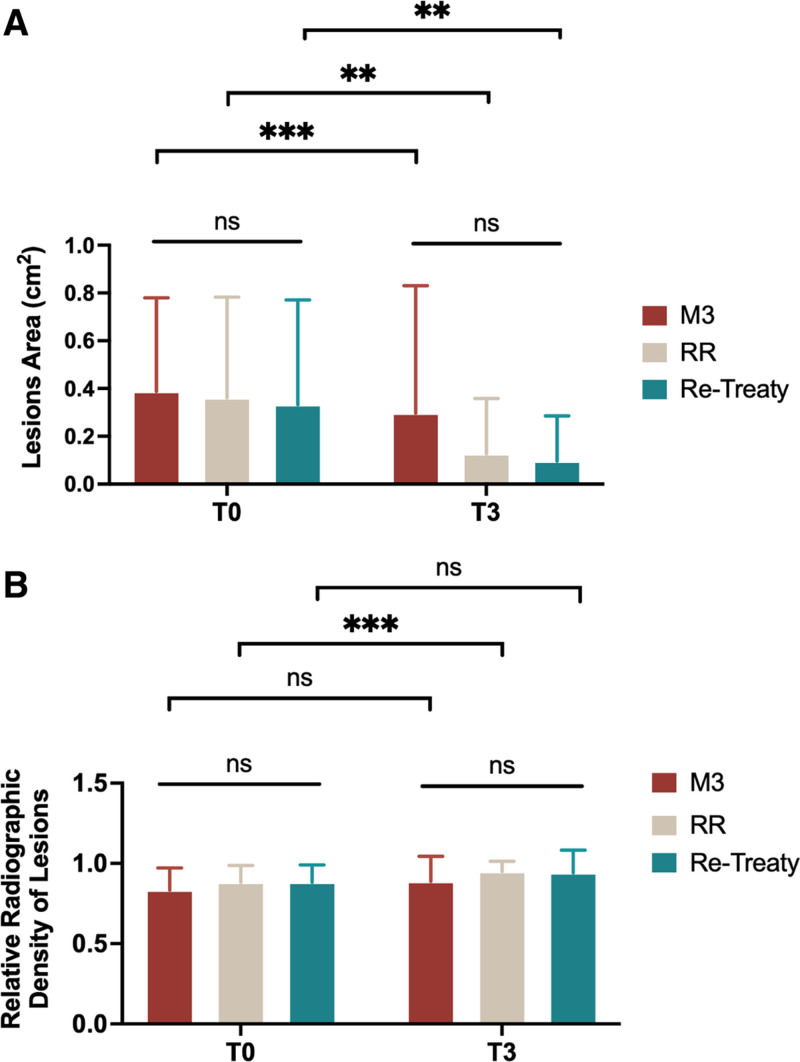
The radiographic analysis. (A) The lesion areas at T0 and T3 were evaluated. While the VAS at T3 was significantly lower than at T0 (*P* < .01), no significant differences were observed among the groups at any time point; (B) the relative radiographic density at T0 and T3 was analyzed. There were no statistically significant differences observed within groups. Values are expressed as means ± SD. **P < .01; ***P < .001. ns = not significant, SD = standard deviation, VAS = visual analog scale.

Table 5 and Figure 3 present the radiographic analysis of the lesion areas and relative radiographic density. Significant reductions in lesion area were observed in all 3 groups from T0 to T3(*P* < .01), indicating that all groups demonstrated substantial changes in lesion size over time. One-way ANOVA revealed no significant differences in lesion area between the groups at T0 (*P* = .9) and T3 (*P* = .086).

**Table 5:** Radiographic analysis.

Lesions Area (cm2) (mean ± SD)	M3	RR	Re-treaty	*P*-value
n	27	27	27	–
T0	0.39 ± 0.39	0.36 ± 0.42	0.33 ± 0.44	.9
T3	0.30 ± 0.53	0.13 ± 0.23	0.10 ± 0.19	.086
*P*-value (paired *t*-test)	<.001*	.004*	<.001*	–
Relative radiographic, density of lesions (mean ± SD)	M3	RR	Re-Treaty	*P*-Value
n	27	27	27	–
T0	0.84 ± 0.14	0.88 ± 0.11	0.88 ± 0.11	.25
T3	0.89 ± 0.16	0.94 ± 0.06	0.95 ± 0.14	.17
*P*-value (paired *t*-test)	.126	<.001*	.076	–

SD = standard deviation.

*P < .05.

For radiographic density, a significant change between T0 and T3 was observed only in the RR group (*P* < .001). However, no statistically significant differences were found between the 3 file systems at either T0 or T3 (Table 5 and Fig. 3).

## 4. Discussion

Numerous studies have assessed the performance of different retreatment file systems and found differences in their in vitro properties compared to traditional systems and to each other, such as the removal efficiency and amount of extruded debris.^[5,7,13–15]^ The extent to which in vitro differences among retreatment files translate into meaningful clinical differences remains underexplored. Given that patients are primarily concerned with pain relief and long-term prognosis rather than the in vitro properties of file systems – such as canal cleaning ability – this study evaluated 2 retreatment file systems (RR and Re-Treaty) and a universal system (M3), focusing on operative time, postoperative pain, and long-term treatment outcomes. Additionally, radiographic healing of lesions was evaluated as an indirect indicator of inflammation.

First, our findings indicate that the RR system required the shortest operative time among the 3 groups, aligning with the findings of Tantiwanichpun, who reported that RR achieved working length 66% faster than other universal nickel-titanium (NiTi) systems, ProTaper NEXT X2.^[5]^ This may be attributed to its active cutting tip, which allows it to penetrate the gutta-percha and reach its working length more quickly.

Additionally, the gutta-percha removal rate is a commonly used indicator for evaluating the performance of files. In 2022, Buranade et al demonstrated that engine-driven nickel-titanium instruments significantly increase material removal rate in the root canal compared to manual H-files.^[8]^

However, the specially designed retreatment NiTi file do not appear to offer an advantage in this particular aspect universal NiTi file. According to Tantiwanichpun, RR has even lower percentage of canal filling removal, particularly in the apical third region (56.32 ± 35.31%), compared to universal file system (93.22 ± 7.39 %).^[5]^ The researcher believed that removal of gutta-percha, especially in the apical third of the root canal, is vital to achieve a satisfactory root canal retreatment outcome. Therefore, residual gutta-percha cones, particularly in the apical third, may serve as a reservoir for necrotic tissue and bacteria, potentially contributing to persistent periapical inflammation and posttreatment disease.^[5,16]^

However, our results do not support this theory because RR system achieved a higher success rate (100 %) at the 1-year follow-up, compared to universal file system (M3: 88.9 %) and another retreatment system (Re-Treaty 92.6 %). Therefore, whether the remaining gutta-percha truly affects the long-term success of RCT remains open to discussion, particularly considering that the root canal has been adequately irrigated with NaOCl and sealed with new filling materials. Further evidence is needed to confirm this hypothesis.

Another noteworthy indicator was the debris extrusion rate. These undesirable debris include necrotic pulp tissue and bacteria, which are related to constant periapical inflammation, inter-appointment flare-ups, and postoperative pain.^[17]^ Gayatri et al reported triple-helix cross-section has significantly more debris (0.3 ± 0.05 g) than S-shaped (0.21 ± 0.06 g) and convex triangular (0.21 ± 0.02 g) cross-sections (*P* < .001). The authors believed that the triple-helix cross-section fitted more closely to the root canal wall, reducing the gap between the file and the canal wall, which prevents sufficient debris from being accommodated along the flutes and consequently leads to its extrusion through the apical foramen.^[18]^

According to Gayatri et al.’ results, S-shaped and convex rectangular cross-sections are considered to have similar debris extrusion capabilities (*P* > .05). However, clinical evidence to support this hypothesis is lacking. To verify this, we conducted VAS and radiographic analyses on M3 (convex rectangular), RR (S-shape), and Re-Treaty (S-shape) to evaluate postoperative pain and periapical healing.

Our clinical trial suggests that no significant difference in VAS scores was observed among the 3 groups, indirectly supporting the conclusion of Gayatri et al.’s in vitro study^[18]^

In addition, our radiographic analysis revealed that all 3 file systems led to a time-dependent reduction in lesion area, which is a commonly used indicator of periapical healing.^[19,20]^ However, the differences among the file systems were not statistically significant. On the other hand, lesion density also did not show differences among groups, but the time-dependent alteration was quite slight. Considering that most patients reported a reduction in symptoms, we believe that radiographic density is not a sensitive indicator of periapical inflammation.

This study had several limitations. First, the VAS was used for pain assessment. The VAS is a commonly used pain measurement tool that is widely applied in clinical research because of its simplicity and ease of use. However, this method has certain limitations: it can only measure the intensity of pain but cannot reflect its qualitative characteristics. Additionally, individual variations in pain perception may lead to differences in scoring. Jensen et al. (2003) suggested that combining the McGill Pain Questionnaire (MPQ) or Short-form MPQ (SF-MPQ) could provide a more comprehensive, multidimensional assessment of pain.^[21]^ Second, the relatively small sample size may have contributed to the lack of significant differences in lesion areas among the groups at 3 months. Third, the short follow-up period limits the evaluation of long-term outcomes; follow-up extending beyond 5 years would provide more robust insights into the stability of root canal retreatment.

In summary, our findings indicate that the RR and Re-Treaty retreatment file systems do not significantly improve the success rate or reduce postoperative pain compared to the universal system (M3). However, the RR system demonstrated a significantly shorter operative time. Reduced chairside time may help alleviate patient anxiety^[22]^ and enhance cost-effectiveness^[23]^ for clinicians.

Future clinical studies should consider increasing the sample size and extending the follow-up period to better evaluate the clinical relevance of different retreatment file systems. Moreover, the variation in treatment outcomes using the same file system across anterior teeth, premolars, and molars warrants further investigation.

## 5. Conclusions

All 3 file systems achieved satisfactory outcomes in root canal retreatment, with no significant differences in the postoperative VAS scores, long-term success rates, or radiographic healing. However, the RR system allows for the faster removal of gutta-percha.

## Author contributions

**Conceptualization:** Qi Fang, Qianpeng Li, Yajun Gao.

**Data curation:** Qi Fang.

**Investigation:** Qi Fang.

**Methodology:** Qi Fang, Qianpeng Li, Yajun Gao.

**Project administration:** Yao Wang.

**Resources:** Yao Wang.

**Software:** Qi Fang.

**Supervision:** Yao Wang.

**Validation:** Qi Fang.

**Visualization:** Qi Fang.

**Writing – original draft:** Qi Fang.

**Writing – review & editing:** Qianpeng Li, Yajun Gao, Yao Wang.
